# Designing a theory- and evidence-based tailored eHealth rehabilitation aftercare program in Germany and the Netherlands: study protocol

**DOI:** 10.1186/1471-2458-13-1081

**Published:** 2013-11-19

**Authors:** Dominique Reinwand, Tim Kuhlmann, Julian Wienert, Hein de Vries, Sonia Lippke

**Affiliations:** 1 CAPHRI, Department of Health Promotion, Maastricht University, P. O. Box 616, Maastricht 6200, MD, the Netherlands; 2 Jacobs Center for Lifelong Learning and Institutional Development, Jacobs University Bremen, Campus Ring 1, 28759 Bremen, Germany

**Keywords:** Cardiac rehabilitation aftercare, Physical exercise, Fruit and vegetable consumption, Multiple behavior changes, Internet based intervention, Tailored feedback, Cardiovascular diseases

## Abstract

**Background:**

Cardiac rehabilitation programs aim to improve health status and to decrease the risk of further cardiac events. Persons undergoing rehabilitation often have difficulties transferring the learned health behaviors into their daily routine after returning home and maybe to work. This includes physical activity as well as fruit and vegetable consumption. Computer-based tailored interventions have been shown to be effective in increasing physical activity as well as fruit and vegetable consumption. The aim of this study is, to support people in transferring these two learned behavior changes and their antecedents into their daily life after cardiac rehabilitation.

**Methods:**

The study will have a randomized controlled design and will be conducted among German and Dutch people who participated in cardiac rehabilitation. The study will consist of one intervention group which will be compared to a waiting list control group. During the eight week duration of the intervention, participants will be invited to participate in the online after-care program once per week. The intervention encourages participants to define individual health behavior goals as well as action, and coping plans to reach these self-determined goals. The effectiveness of the program will be compared between the intervention condition and the control group in terms of behavior change, antecedents of behavior change (e.g., self-efficacy), ability to return to work and increased well-being. Further, subgroup-differences will be assessed including differences between the two countries, socioeconomic inequalities and across age groups.

**Discussion:**

The present study will make a contribution to understanding how such an online-based tailored interventions enables study participants to adopt and maintain a healthy lifestyle. Implications can include how such an online program could enrich cardiac rehabilitation aftercare further.

**Trial registration:**

NTR 3706, NCT01909349

## Background

Cardiovascular diseases (CVDs) are a major health problem in western countries and cause approximately 30% of the global deaths [1,2] and 48% of the deaths in the EU [3]. Medical cardiac rehabilitation is a multifaceted approach to improve social, mental and physical recovery from any cardiac event. This rehabilitation aims at preventing future events by reducing CVD risk behaviors such as insufficient physical activity and unhealthy nutrition patterns [4]. Regular physical activity and healthy nutrition habits are important components within rehabilitation treatment [5], as these lifestyle changes reduce mortality and morbidity in CVD cases [6,7].

Physical activity has a wide range of beneficial effects and has been proven to be protective against several diseases such as hypertension, type 2 diabetes mellitus and obesity [8-10]. As a result of regular physical activity patients in rehabilitation gain a better physical condition, muscle strength and report a reduction of pain [11,12]. Studies also reveal that CVD patients have reduced mortality and morbidity if they improve their physical condition [13,14]. Furthermore, maintenance of physical activity is associated with a 45% risk reduction for subsequent CVDs [15].

The benefits of a diet rich in fruit and vegetables have been extensively studied and revealed that it reduces the risk of future cardiovascular issues and lowers CVD mortality by approximately 27% [16-20]. Furthermore, fruit and vegetable consumption reduces the risk factors for cardio vascular diseases like high cholesterol levels [21]. A healthy diet increases health (e.g., via a reduction of body weight) and is therefore important for people undergoing rehabilitation [22,23].

Even though several studies have demonstrated that adhering to rehabilitation treatment will reduce medical costs [24] and reduce morbidity and mortality by 20% to 25% [25], it is difficult for many patients in rehabilitation to adopt and especially maintain healthy behaviors after they participated in rehabilitation programs [26-28].

Because of these health benefits, the maintenance of a healthy lifestyle is important for those patients beyond the duration of the rehabilitation treatment. Yet, aftercare programs are limited and not always available for cardiac rehabilitation patients [29]. Computer-based tailored health interventions are a promising tool for multiple behavior change and provide several benefits: patients can take part in the aftercare from home and do not have to travel; patients can participate at their preferred time and it is cost-effective because more people can be reached compared to face-to-face or group aftercare programs [29-32]. Due to simplicity and ease of use online-based programs promise to be effective in implementing the new behavior changes into daily routines. Computer-based programs also provide the opportunity to deliver tailored messages. Tailored feedback is more effective than generic messages which provide standardized information [33,34] and also more cost effective [35,36]. Current studies have shown promising results in raising the motivation of patients in rehabilitation to increase fruit and vegetable consumption and physical activity [37,38]. However, these previous studies have not made use of computer based personalized feedback which might increase their effectiveness.

This personalized information is based on individual assessment, meets individual needs and contains less redundant information. Therefore personalized information is remembered better and increases the intention to change behavior [39-41]. Tailored messages should include different kinds of feedback: personal behavioral feedback and action feedback to increase the effectiveness [33]. Personal feedback provides the person with a reflection of the actual behavior [42]. This feedback mechanism increases the risk perception, awareness and the motivation to change [43,44]. Action feedback provides the individual with practical information about how to change and maintain certain behaviors [45]. Tailoring can be categorized into two broad forms: static and dynamic. Static tailoring is based on one assessment at the beginning of the intervention while dynamic tailoring can be done by assessing the information needed at the relevant time before the feedback is given. Dynamic tailoring was shown to be more effective in health behavior change interventions [34,41].

This research project will be conducted and evaluated in the Netherlands and Germany to investigate if an online-based rehabilitation aftercare program is equally effective in countries with different rehabilitation systems.

In the Netherlands, the majority of cardiac patients receive aftercare from their general practitioner (GP) after discharge from hospital. Only a small percentage of CVD patients (28%) receive rehabilitation [46]. Most of these patients receive rehabilitation in policlinics (80%), a minor part is treated in rehabilitation centers (20%) [5]. The Netherlands have a dense network of medical facilities working together (hospitals, policlinics, rehabilitation centers, medical practices) enabling patients to reach cardiac rehabilitation without long travel distances [47].

In Germany, the number of patients who receive cardiac rehabilitation is low as well. Only one third of cardiac patients are transferred to rehabilitative treatment [48]. However, in contrast to the Netherlands, rehabilitation in Germany mainly takes place as an inpatient service and patients are seldom able to stay at home during partaking the rehabilitation treatment [49,50]. This implies that, in contrast to the Netherlands, a major part of the patient education needs to be provided in the clinical setting. In the Netherlands the majority of persons undergoing rehabilitation is treated within policlinics for several hours a week and can stay in their home environment. Besides, the concept of aftercare in Germany differs from that in the Netherlands. In Germany, CVD patients are offered intensive aftercare rehab (IRENA), intensive after care (INA) and cardiac exercise groups with the aim of reintegration into the work life [48]. This aftercare is not provided in the Netherlands [51]. How these structural differences work together or boost the effect of an online intervention addressing health behavior adoption and maintenance was not tested before and is the main unique feature of this study.

### Study aims

The aim of the present study is to test the effectiveness of the online-based aftercare program “RENATA (Rehabilitation aftercare program for an optimal transfer into daily life)” for cardiac rehabilitation in order to improve physical activity and fruit and vegetable consumption. To acquire a deeper understanding of the usefulness of aftercare interventions in countries with different rehabilitation systems, the study will be implemented in Germany and the Netherlands.

## Methods

### Ethical approval

The study protocol was approved by the Medical Ethics Committee of Atrium Medical Centre Heerlen in the Netherlands (METC number 12-N-124) and by the *Deutsche Gesellschaft für Psychologie* (DGPs; EK-A-SL 022013). The study was registered with ClinicalTrials.gov (Identifier: NCT01909349).

### Study design

This multinational and multicenter longitudinal study has a quasi-experimental randomized controlled trial design. The study consists of one intervention group (IG) and one waiting list control group. The intervention group will first address physical activity then fruit and vegetable consumption. The waiting list control group will get access to the intervention after the study condition has finished the intervention (T3). The waiting list control group has to complete the baseline questionnaire (T1) at the same time as the intervention group and will have access to the intervention only after their second measurement. In total, six data collection time points are planned where T0 is intended for the collection of contact information only. Baseline measurement will take place at T1 before the intervention starts, followed by a measurement (T2) after completion of the intervention. Further follow-up measurements are planned to take place after four weeks (T3), after six months (T4) and after 12 months (T5) (see Table 1).

**Table 1:** Design of measurement points

**Time point**	**Measurement**
T0	Patients within cardiac rehabilitation will be asked if they are interested in participation of an aftercare program. Contact data will be collected
	**End of rehabilitation and start intervention**
T1	After patients have completed rehabilitation the intervention starts
T2	After 8 weeks the intervention is completed
	**End of the intervention period**
	**Waiting list control group starts intervention**
T3	4 week follow-up measurement
T4	6 month follow-up measurement
T5	12 month follow-up measurements

### Participants and recruitment

Participants will be recruited in cardiac rehabilitation facilities in Germany and the Netherlands*.* The study population will consist of people aged up to 85 years who successfully completed cardiac rehabilitation. In order to be eligible to participate in this study participants need to be able to use a computer, familiar with the use of the Internet, and need sufficient reading and writing language skills in the relevant language (German or Dutch). Patients with contraindications for physical activity and fruit and vegetable consumption will be excluded (e.g., heart failure patients which are not allowed to eat 2 pieces of fruit per day [5]).

### Power analysis

The power analysis was based on the assumption that the intervention will result in a small improvement of effect to improve fruit and vegetable consumption and in a small to medium effect to improve physical activity for the study conditions [38]. The calculation was based on an effect size of 0.3, with a power (1-β) of 0.8 and alpha of 0.05.

No cluster effect is expected due to the fact that the randomization of the participants is independent of the place of recruitment. Further, it is necessary to take interaction effects into account between the intervention and the control group, and between Germany and the Netherlands.

Therefore, to be able to detect a small to medium effect, 263 people have to participate in every group in each country. This means that the required total sample size will need to consist of 1052 participants, not adjusted for potential drop out during the intervention.

### Randomization

During the rehabilitation period, patients will be recruited within the rehabilitation center by their therapists. These therapists will first inform the person undergoing rehabilitation about the study and hand out study information. Participants will log in on the project website and have to give online informed consent. Randomization will be done by the computer software *TailorBuilder*, which is developed for internet based tailored interventions. Participants will be randomly allocated to the study group or to the waiting list control group. The waiting list control group will get access to the intervention after eight weeks (see Table 1).

### Intervention

The Health-Action Process Approach (HAPA) will be used as a theoretical framework to develop the intervention [52,53]. The HAPA Model distinguishes two phases during the process of behavior change: a motivational phase in which the intention to perform a specific behavior is formed and a volitional phase in which the intention is translated into the actual performance of the behavior. Depending on the stage, people have different needs. Within the motivational phase positive outcome expectancies, a high risk perception and a high self-efficacy to perform the specific behavior are necessary to develop an intention. Once people have formed the intention to perform a certain behavior, skills such as action and coping planning, self-efficacy, and social support are necessary. The HAPA Model further distinguishes between stages of intention. Non-intenders are in the motivational phase and have no intention to change their behavior. Intenders are in the volitional phase but do not perform the behavior in contrast to actors, who carry out the behavior [52]. The intervention will target the concepts of the different stages via the use of behavior change techniques. In line with Abraham and Michie [54], we use several behavior change techniques like providing information about behavioral risk and benefit of behavior change, prompting intention formation, prompting barrier identification, providing instructions how to perform a behavior, prompting specific goal setting and review of behavioral goals, providing feedback on performance, prompting practice and providing follow-up prompts, prompting to plan social support and finally prompting relapse prevention, also based on strategies used by other effective computer tailoring programs [55,56].

During the eight-week intervention period, participants will be invited to access the online program once per week and take part in one of the intervention modules. These eight modules aim to increase participants´ risk perception of CVD, support positive outcome expectancies towards physical activity and fruit and vegetable consumption, and guide participants in defining and reflecting on their own goals, action plans and coping plans. Further, self-efficacy regarding the participant’s ability to perform and maintain the two targeted behaviors will be addressed.

Table 2 presents a short overview about the weekly intervention content. During the first session, participants will receive feedback and information about their risk perception, outcome expectancies and their behavior with regard to physical activity and fruit and vegetable consumption which is based on the previous assessment. During the following session, participants formulate their own individual goals and specific plans with regard to physical activity and fruit and vegetable intake. Participants will receive information about how to structure a plan as good as possible. For example *“I would like to go for a walk in the park for 30 minutes each Tuesday.”* All plans have to be evaluated with regard to their feasibility through self-reflection. This will be achieved by asking the participants questions like: *“Is your plan ‘to go for a walk in the park’ easy to implement into your daily routine?*”

**Table 2:** Intervention content

**Weekly sessions**	**Weekly content**
Session 1 PA and Session 5 F&V	Questionnaire and personalized feedback, increase risk perception and outcome expectations, defining own health outcomes; (Session 5: Assessment of social cognitive factors)
Session 2 PA and Session 6 F&V	Personalized feedback, defining action plans
Session 3 PA and Session 7 F&V	Personalized feedback
	Evaluation and self-reflection about action plans
	Adjusting action plans
	Defining personal barriers
	Develop personal coping plans
	Evaluation and refection about coping plans
Session 4 PA and Session 8 F&V	Personalized feedback, adjusting coping plans, thinking about social support and development of a list of potential supporters from the social environment; (Session 8: T2 questionnaire)

**(PA)** physical activity; **(F&V)** fruit and vegetable consumption.

After reviewing the plans, participants get the opportunity to adjust them. After each session, participants will be motivated to practice their plans. During the next session, participants are asked to evaluate their action plans and if necessary adjust them. People who indicate to have problems formulating plans will receive role model examples. Furthermore, they are asked to identify their personal barriers and obstacles that prevent them from putting plans into practice. Barriers need to be defined and participants are again asked to define plans on how to deal with those plans. For example, if someone chooses “*It is difficult for me to eat fruit and vegetables when I am not at home*”, the person will be asked to think about a strategy to be able to eat fruit and vegetable like *“when I leave the house for some hours I will take an apple with me”*. Defining action and coping plans has been proven to be an effective tool to increase physical activity among cardiac rehabilitation patients [57]. Again, these coping plans have to be evaluated during the next session and can be adjusted if necessary. During the last session of each behavior, participants will be asked to think about persons in their social environment who could be able to support their change and can be useful to help them.

The intervention will be enriched by different kinds of feedback: ipsative feedback will be used to provide participants with an overview of their development with regard to physical activity and fruit and vegetable consumption [41,58,59]. At the beginning of each session participants will be asked about the physical activity or fruit and vegetable consumption during the preceding seven days. After that, they will receive tailored feedback about their behavior.

**Figure 1:**
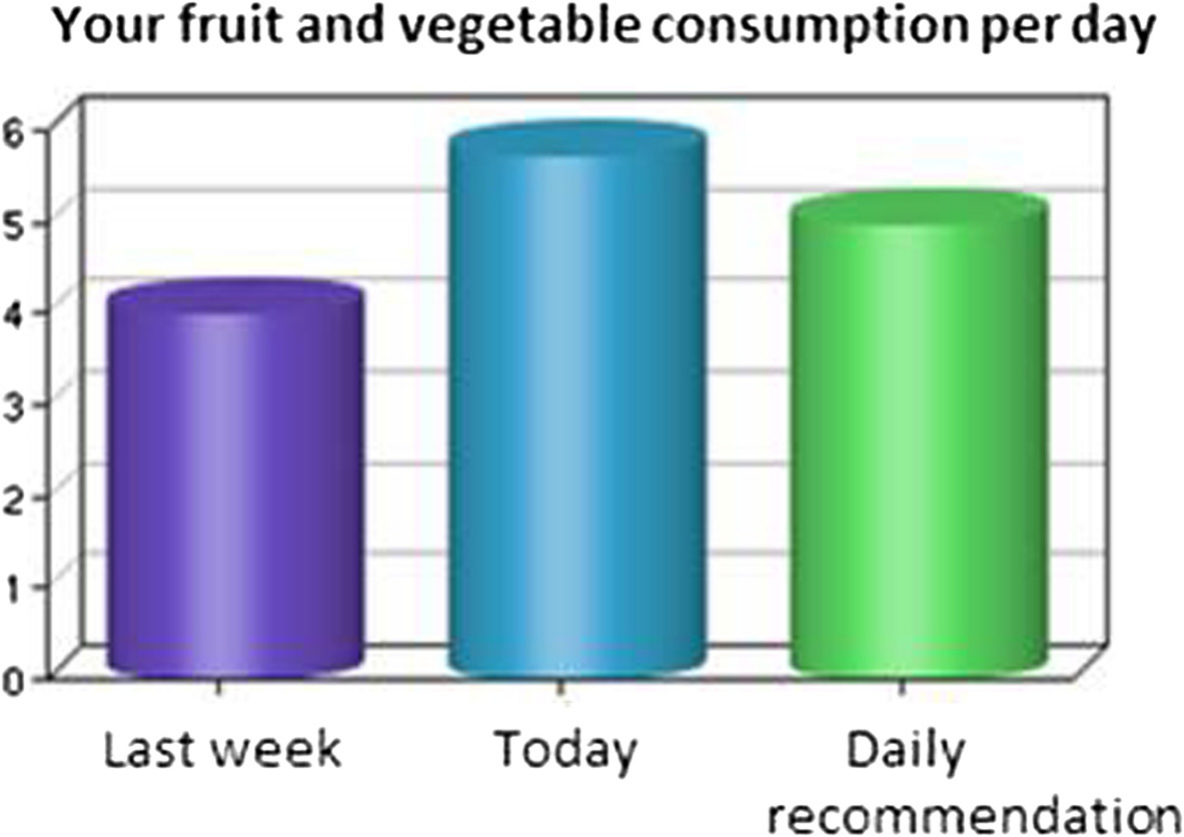
Ipsative feedback pieces of fruit and vegetable consumption.

Normative feedback will be provided to give information about whether the behavior meets the recommendations for both target behaviors [42,56]. Feedback will not only be based on the assessment at the beginning of the intervention T1 but also given dynamically with the use of information that will be assessed during the intervention. In this way the feedback is based on the newest information and contains more relevant information. *“Ben, you ate 3 portions of fruit and vegetable per day. This is a bit more than the last time. Great! But remember, it would be good for you to eat at least 5 portions fruit and vegetable a day. RENATA can support you to achieve this goal.”* In addition, a figure (see Figure 1) will present the prior behavior, the behavior of the past weeks, and whether participants fulfill the recommended amount of physical activity or fruit and vegetable consumption.

### Measurement instruments

If not mentioned otherwise, all items will be measured with the use of visual analogue scales (VAS). These scales have several advantages in comparison with Likert-scales: participants need less explanation to answer question, nuancing of responses is more exactly, drop-out rates are lower in and VAS allows for grater statistical analysis [60,61].

### Demographic and socioeconomic characteristics

Several items address the demographic and socioeconomic characteristics. These include gender, age, level of education, family status, living conditions, country of origin, native language and employment status.

### Health status

Health status will be measured by asking the reasons for the cardiac rehabilitation, body height and body weight.

### Past health behavior

The level of physical activity will be assessed with the use of the short version of the International Physical Activity Questionnaire (IPAQ) [62]. Participants will be asked to estimate how many days during the past seven day they were vigorous and moderate physically active. Further, the amount of days and spend time for walking during the past seven days will be asked. The IPAQ is a valid measurement instrument which allows an international comparison of participant’s physical activity level.

To assess the fruit and vegetable consumption during the past seven days, four items will be used. Participants will be asked to count the number of consumed portions or glasses fruit and vegetable a day. It will be distinguished between fruit, fruit juice/vegetable juice, cooked or steamed vegetable, and salad and raw vegetable [63].

### Intention

Intention to perform physical activity will be measured with the use of three items on a VAS scale ranging from *not true* to *exactly true*. These items are: “*On 5 days a week for 30 minutes (or a minimum of 2.5 hours per week), I have the intention to …” “…perform strenuous physical activity (heart beats faster, sweating)”. “…be moderately physically active (not fatiguing, mild sweating)*” and “…*be moderately physically active (not fatiguing, mild sweating)*” [64].

Intention about fruit and vegetable consumption will also be measured with three items: *“I seriously intent to …” “… eat at least 5 portions of fruit and vegetable daily “, “…eat fruit and vegetable at every meal” and “drink at least one fruit or vegetable juice every day*” [64].

### Stages of change

The stage of change for physical activity will be assessed with the item: *“Please think about your typical weeks: Did you engage in physical activity at least 5 days per week for at least 30 minutes?” *[64]. Regarding dietary behavior, participants will be asked *“Please think about what you have typically consumed during the last weeks: Did you eat five portions of fruit and vegetables per day?”* Responses are based on a rating scale with verbal anchors *“No, and I do not intend to start”*; “*No, but I am considering it”*; *“No, but I seriously intend to start”*; *“Yes, but only for a brief period of time*”; *“Yes, and for a long period of time”*.

### Self-efficacy

The concept of self-efficacy will be measured in three parts; motivational self-efficacy refers to one’s confidence to perform a specific behavior. Maintenance self-efficacy refers to one’s confidence to perform a specific behavior over a long period of time and recovery self-efficacy refers to one’s confidence to resume a specific behavior after a discontinuation [52].

Motivational self-efficacy will be assessed with one item for physical activity: *“I am certain that I can be physical active a minimum of 5 days a week for 30 minutes even it is difficult.”* And also with one item for fruit and vegetable consumption: *“I am certain that I can eat at least 5 portions of fruit and vegetables a day even if it is difficult”*[65].

Maintenance self-efficacy will be assessed with two items for each target behavior. *“I am certain that I can be physically active permanently at a minimum of 5 days a week for 30 minutes …* / *“I am certain that I can permanently eat 5 portions of fruit and vegetable a day…” “… even if it takes a lot of time till I am used to do it”* and *“… even if I have worries and problems”*[66].

Recovery self-efficacy will be measured with two items for each behavior: *“I am certain that I can again be physically active a minimum of 5 days a week for 30 minutes / I am certain that I can again eat 5 portions of fruit and vegetables a day …” “even if I changed my concrete plans several times”* and *“… even if I skipped a few times”*[66]. Participants can answer all self-efficacy items on a VAS-scale ranging from *don’t agree at all* to *agree completely*.

### Outcome expectancies

Participants’ outcome expectancies will be measured by four items; two about positive and two about negative expectancies. *“If I am physical active 5 days a week for at least 30 minutes, then… ”… this is good for my health”*, *”…I feel better afterwards”*, *”…it will cost me a lot of time”* and *“…this will be a financial burden”* . The items for fruit and vegetable consumption: *“If I daily eat 5 portions of fruit and vegetable, then…” “… this is good for my health”*, *“…I feel better afterwards”*, *“…it will cost me a lot of time”* and *“…this will be a financial burden”*[67]. Answers ranging from *don’t agree at all* to *agree completely*.

Also outcome expectations regarding fruit and vegetable consumption will be assessed with six items: “If I consume at least 5 portions of fruit and vegetable a day, then …” “… this good for my health”, *“…I feel better afterwards”, “… I get more energy”, “…it will cost me a lot of time”*, *“…this will be a financial burden”* and *“… this will cost a lot of effort”. Answers can be given on a* VAS-scale (*totally disagree* to *totally agree).*

### Risk perception

To assess participants risk perception an adaption of Perloff’s and Fetzer’s [68] perceived vulnerability scale will be used: *“How likely is it that you will have a sometime in your life …” “… a high cholesterol level?”*, *“… a heart attack?”*, “…*a high blood pressure?”*, *“… a stroke?”* and *“… a cardiovascular disease?”*. Answers can be given on a VAS-scale: *unlikely to very likely*.

### Action plans

Action planning will be assessed with the use of three items for physical activity and with three items for fruit and vegetable consumption. For both target behaviors the question starts with: “*For the next month I already planned in detail …” “… which physical activities I would like to do.” “… where I will be physical active” and“… on which days I will be physical active”*[69].

Fruit and vegetable consumption will start with the same beginning following with these three items: *“…when I will eat 5 portions fruit and vegetable”, “which fruit and vegetable I will eat”,* and *“… how I will prepare the food”*. Participants can agree or disagree on a VAS-scale [69].

### Coping plans

If and to what degree participants have made coping plans will be measured with three items for each of the two target behaviors. Physical activity: “*For the next month I already planned in detail …” “… when I have to be especially cautious not to stop being active”, “… what I can do in difficult situations to stick to my intentions”* and “*… how I continue to stay active even when something comes in between”*. Fruit and vegetable consumption will have the following items *“… when I need to be especially cautious not to fall into my old eating habits”, “… what I can do in difficult situations to stick to my intentions”* and “*… how I continue to eat healthy even when something comes in between”*. Answers can be given on a VAS-scale (*don’t agree at all* to *agree completely*) [69].

### Social support

Perceived social support will be assessed with three items for each behavior [70,71]. For physical activity the items are: “My partner helps me/My family helps me/My circle of friends and acquaintances help me… to be me to be physical active for at least 30 min. on 5 days a week (or a minimum of 2.5 hrs. per week)”. Perceived social support for fruit and vegetable consumption will be assessed with: “My partner helps me/My family helps me/My circle of friends and acquaintances help me… to eat 5 portions of fruit and vegetable per day”.

### Habit

The habituation of physical activity and fruit and vegetable consumption will be measured with an abbreviated version of the Self Report Habit Index (SRHI) [72]. *“Being physically active for at least 30 minutes on 5 days a week is something that…”* and *“Eating 5 portions of fruit and vegetable per day is something …” “… has become a confirmed habit”* and *“… I do without thinking about it”*. Answer can be given on a VAS-scale ranging from *do not agree* to *completely agree*.

### Quality of life

Quality of life will be determined by means of the World Health Organization Quality of Life-BREF (WHOQoL-BREF) questionnaire [73,74]. This instrument was developed to assess quality of life in a cross-culturally comparable way. General quality of life will be measured via the question: “How would you rate your quality of life?” with an answer category from *very poor* to *very good*. The physical health subdomain with seven items will also be used. Examples of the physical health items are: “To what extent do you feel that physical pain prevents you from doing what you need to do?”, “How much do you need any medical treatment to function in your daily life?” and “How satisfied are you with your ability to perform your daily living activities?”

### Depression

The level of depression will be measured using the Center for Epidemiologic Studies Short Depression Scale (CES-D 10) [75]. The ten items from this scale are: In the past week … “*I was bothered by things that usually don’t bother me“, “I had trouble keeping my mind on what I was doing”, “I felt depressed”, “I felt that everything I did was an effort”, “I felt hopeful about the future”, “I felt fearful”, “My sleep was restless”, “I was happy”, “I felt lonely” and “I could not get going*”. Answers can be given on a 4-point Likert scale ranging from *rarely or none of the time (less than 1 day)* to *most or all of the time (5–7 days)*.

### Subjective age

Based on Boehmer [76], two items will be asked to assess the subjective age of the respondents. *“How old do you feel physically?*” and “*How old do you feel mentally?”*

### Compensatory health beliefs

The scales used to access compensatory health beliefs concerning physical activity and fruit and vegetable consumption will be based on Knäuper and colleagues [77]. The scale contains four items such as: “*Too little physical activity can be compensated by eating less”*. Answers are indicated on a VAS-scale (*don’t agree at all* to *agree completely*).

### Main study outcomes

The primary study parameter of this study is to analyze the effectiveness of a rehabilitation aftercare program with regard to the level of physical activity and fruit and vegetable intake. The effect of the program will be compared between the intervention group and the control group as well as between the different countries. Secondary study parameters that will be investigated are self-efficacy, outcome expectations, habit, compensatory cognitions, social support, action and coping planning, intention to change, duration absence from work, quality of life and wellbeing. The other study parameters which will be considered are demographic variables, socioeconomic status, stage, age, gender, depression, and health status.

### Statistical analysis

Descriptive statistics will be used to describe baseline characteristics of the study. We will assess whether there are significant differences between the intervention group in comparison with the control group according to physical activity and nutrition by means of linear regression analysis in PASW (SPSS). The pre-test and post-test measurements of the control group will be compared with the measurements of the intervention and controle group with regard to the target behaviours.

To be able to assess the impact of the intervention on the performance of physical activity and fruit and vegetable consumption, we intend to perform several analyses of covariance (ANCOVA) after four weeks (T3) and six months (T4), controlling for the baseline performance. Multivariate regression analysis will be used to determine possible confounding or modification effects. Regression analysis will allow us to identify the percentage of variability of the dependent variable that is explained by the determinants used. In order to detect differences between the study condition and the control group, effect sizes will be calculated for each study group separately [78].

Subgroup analyses will be performed to determine if the benefit of the intervention depends between groups like nationality or socioeconomic status [79]. Further, dose–response-analyses will be conducted with logistic regression to see if the intervention effect depended on the time spent on the webpage.

Baseline characteristics of participants who dropped out will be compared with participants who finished the intervention to examine whether drop out is at random or determined by specific characteristics.

## Discussion

The results of this study are important for the future development of online-based rehabilitation aftercare programs for cardiovascular patients and the development of online interventions in general. Although there is no doubt about the positive effect of adequate physical activity and fruit and vegetable consumption for people undergoing rehabilitation [8,11,13,17,22,80], many patients fail to maintain lifestyle changes [26-28]. To transfer the rehabilitation results into the daily life and to overcome unhealthy habits, effective rehabilitation aftercare is necessary.

Given the benefit of tailored health behavior change interventions, there is still a need for further research. It is important to develop guidelines for interventions which are effective in different countries with different rehabilitation systems. In order to make a positive contribution, an international implementation and evaluation of this study will be done within Germany and the Netherlands.

It will be assessed to what extent the health status, quality of life and return to the labor market can be realized among the participants. Our hypothesis is that participants in the intervention group will increase their level of physical activity, fruit and vegetable consumption and are able to maintain these behaviors over a longer period of time compared to the control group.

## Competing interests

All authors declare that they have no competing interests. Hein de Vries is also scientific director of Vision2Health, a company aimed at implementing evidence based eHealth programs.

## Authors’ contributions

SL developed the study concept and aims. DR, JW and TK designed the intervention, SL and HdV provided intellectual input for the development of the questionnaire and intervention. DR drafted the manuscript, JW, TK, SL and HdV provided extensive feedback on the manuscript. All authors read and approved the final manuscript.

## Pre-publication history

The pre-publication history for this paper can be accessed here:

http://www.biomedcentral.com/1471-2458/13/1081/prepub
